# Supplementary material for: The conserved ATPase PCH-2 controls the number and distribution of crossovers by antagonizing their formation in Caenorhabditis elegans
Source: eLife. 2025 Feb 18;13:RP102409. doi: 10.7554/eLife.102409 (PMC11835387; doi:10.7554/eLife.102409)
Supplement: Supplementary file 1. — SNPs, primers, enzymes used for restriction digests, and expected fragment sizes used for the recombination assay. [file elife-102409-supp1.docx]

**Supplementary Table 1: Single nucleotide polymorphisms (SNPs) used for recombination assay**

| Chromosome and SNP | Primer Name | Primer Genetic  Location | Primer  Sequence  FOR | Primer  Sequence  REV | Restriction enzyme | N2 | HI |
| --- | --- | --- | --- | --- | --- | --- | --- |
| IA | F56C11 | -19 | ATGCCAGTGATAAGGAACGG | TCACATCCCTTGTCGATGAA | DraI | 354, 146 | 500 |
| IB | Y71G12 | –12.3 | GACAATGACCAATAAGACG | GATCCGTGAAATTGTTCCG | BsrI | 440, 125 | 364, 125, 76 |
| IC | K04F10 | 0.9 | ATCATTCTCCAGGCCACGTTAC | CTGAACTAGTCGAACAAACCCC | NdeI | 594 | 300, 294 |
| ID | T07D10 | 13.6 | CTTGGTGTGGGGAGAGTATAGG | TTTGTCCGGATTGACTCTGC | Sau3AI | 303, 63 | 207, 96, 63 |
| IE | ZK909 | 28.8 | CACAAGTGGTTTGGAAGTACCG | CAACAAAGGGATAGATCACGGG | HindIII | 450 | 236, 214 |
| IIIA | pkP3081 | -26.98 | AGCAAGAATGAGCCGATTG | GTCGGCCGTTTTCAAATAACTG | TaqI | 222, 149 | 195, 145, 27 |
| IIIB | pkP3095 | -5.12 | TCTCGTCAATTGTCGCCTG | TTATTTGCAATCCAACGGC | ApoI | 308 | 168, 140 |
| IIIC | pkP3101 | -0.9 | CCAAGTGCAAACTATGGTGC | ATAAACAATTTCAGTGCCGC | HinfI | 495 | 282, 213 |
| IIID | pkP3035 | 0.9 | CGTAAACTACCAAACTCGGTG | GGTCTACTACAACTATACAGGC | Eco0109I | 732 | 419, 313 |
| IIIE | pkP3080 | 21.29 | CGGTGGTGGTAAAAGTGTAAC | CAACATTCAGGCTGTGCTTTCC | Hpy188III | 365, 76, 68, 35 | 241, 124, 76, 68, 35 |
| IVA | F56B3 | -24 | TGATGGTGTGTCTGCGTACC | AGAGCTGGAGAGCACGGATA | DraI | 301, 128, 71 | 429, 71 |
| IVB | F52C12 | −14.9 | ACATTTAGTCACGCGTAGGG | GCCCGAATCTAGCACATAAG | HpaII | 191, 137, 22 | 328, 22 |
| IVC | B0273 | 1.8 | AATACAGCAGTCGTTCCGTTC | TGAACTTCATGAACCAGCTTG | DraI | 288, 144 | 432 |
| IVD | K10D11 | 6.7 | GATTATTTCAGAGGAGCAGAGC | CATAGCACGTGGAATAACCAC | HindIII | 420 | 245, 175 |
| IVE | T02D1 | 16.8 | TGCTTAAAGTCATCGTGTCCAC | TGTAAACCGTATCGAATCCGAC | EarI | 174, 235 | 408 |
| XA | pkP6139 | -19.97 | AAGAGTGAACCTTTTCCGTGAG | TGATGCAATTTATACACACGCC | MseI | 401, 31 | 279, 122, 31 |
| XB | pkP6120 | -10.46 | TCGTGGCACCATAAAAGTG | GATTCAGATCAAACAGAGGTGG | DraI | 243 | 128, 115 |
| XC | pkP6157 | -0.14 | GGGGTATAATGAACCAACCTG | TGTAGGAACCGTTTGTTTCTTC | ApoI | 261, 48 | 150, 111, 48 |
| XD | pkP6161 | 9.38 | ATCGACCCCAACAATGCAC | TCCGTCATCCAAATCTCCG | AseI | 542 | 287, 255 |
| XE | pkP6170 | 24.07 | CGCTGTCACAATCTCTAAAATG | AAACCCTCCCCACTTTGTTGTC | ApoI | 249, 118, 56 | 197, 118, 56, 52 |
